# Detection and characterization of zoonotic pathogens of free-ranging non-human primates from Zambia

**DOI:** 10.1186/s13071-014-0490-x

**Published:** 2014-10-29

**Authors:** Jesca Nakayima, Kyoko Hayashida, Ryo Nakao, Akihiro Ishii, Hirohito Ogawa, Ichiro Nakamura, Ladslav Moonga, Bernard M Hang’ombe, Aaron S Mweene, Yuka Thomas, Yasuko Orba, Hirofumi Sawa, Chihiro Sugimoto

**Affiliations:** Division of Collaboration and Education, Research Center for Zoonosis Control, Hokkaido University, Kita 20, Nishi 10, Kita-ku, Sapporo, Hokkaido, 001-0020 Japan; National Livestock Resources Research Institute (NaLIRRI), P.O. Box 96, Tororo, Uganda; Unit of Risk Analysis and Management, Research Center for Zoonosis Control, Hokkaido University, Kita 20, Nishi 10, Kita-ku, Sapporo, Hokkaido, 001-0020 Japan; Hokudai Center for Zoonosis Control in Zambia, School of Veterinary Medicine, University of Zambia, PO Box 32379 Lusaka, Zambia; Department of Paraclinical Studies, School of Veterinary Medicine, University of Zambia, PO Box 32379 Lusaka, Zambia; Department of Disease Control, School of Veterinary Medicine, University of Zambia, PO Box 32379, Lusaka, Zambia; Division of Molecular Pathobiology, Research Center for Zoonosis Control, Hokkaido University, N20, W10, Kita-ku, Sapporo, 001-0020 Japan

**Keywords:** Non-human primates, Reservoir, Pathogens, Zoonosis, Zambia

## Abstract

**Background:**

Wildlife may harbor infectious pathogens that are of zoonotic concern acting as a reservoir of diseases transmissible to humans and domestic animals. This is due to human-wildlife conflicts that have become more frequent and severe over recent decades, competition for the available natural habitats and resources leading to increased human encroachment on previously wild and uninhabited areas.

**Methods:**

A total of 88 spleen DNA samples from baboons and vervet monkeys from Zambia were tested for zoonotic pathogens using genus or species-specific PCR. The amplified products were then subjected to sequencing analysis.

**Results:**

We detected three different pathogenic agents, including *Anaplasma phagocytophilum* in 12 samples (13.6%), *Rickettsia* spp*.* in 35 samples (39.8%) and *Babesia* spp*.* in 2 samples (2.3%).

**Conclusion:**

The continuously increasing contacts between humans and primate populations raise concerns about transmission of pathogens between these groups. Therefore, increased medical and public awareness and public health surveillance support will be required to detect and control infections caused by these agents at the interface between humans and wildlife.

## Background

Wildlife poses a threat as a potential source of emerging infectious diseases (EIDs) to biodiversity conservation as well as human health. Three quarters of zoonotic EIDs are caused by pathogens in wildlife and the incidence of such diseases is increasing significantly in humans [1,2]. Human activities have contributed to a closer contact between humans and wildlife due to a complex relationship between social and environmental factors causing a major threat both to human health and biodiversity conservation mainly through disease transmission between the two groups [3-5].

The Order Primates has traditionally been divided into two main groupings: prosimians and anthropoids (simians). Non-human primates (NHPs) are a diverse group of animals. Generally, Old World monkeys (Catirrhini) and apes (Hominoidea) are those found in Africa, the Indian sub-continent and in East Asia. New World or neotropical NHPs (Platirrhini) are found in South and Central America. In Zambia, baboons and vervet monkeys are the major non-human primates not only in wildlife management regions, but even out of the management areas. Human-monkey conflicts in the form of crop damage, grabbing of personal effects and direct injury are reported [6].

Several hundred infectious diseases are classified as zoonotic diseases as they are caused by bacteria, viruses, fungi, prions or parasites that can be transmitted from animals to humans and vice versa [7]. Transmission can be direct or indirect, via another organism, either a vector or an intermediate host. Invertebrates spread pathogens by two main mechanisms, either through their bite, or their feces, thus, transmission occurring mechanically or biologically. Tick-borne microbial pathogens, which cause human and zoonotic diseases such as Lyme disease, anaplasmosis, ehrlichiosis, babesiosis, Q ("query") fever, tick-borne encephalitis, Crimean–Congo hemorrhagic fever, Rocky Mountain spotted fever, Colorado tick fever, tick typhus and tularemia, have enormous negative impacts on human health and economic development worldwide. Other zoonotic disease vectors include tsetse flies (*Glossina* spp*.*) transmitting trypanosomiasis, sand flies (*Phlebotomus* spp.) transmitting leishmaniasis and mosquitoes (*Culicidae* spp.) transmitting malaria.

The hotspots of zoonotic disease transmission include livestock markets, urban and peri-urban wildlife and farming on fragments and edges of wildlife conservation areas and buffer zones. We hypothesized that a possible interaction between human and simian pathogens coming from a zoonotic cycle cannot be disregarded because simians that live in the areas of the disease endemic foci of Africa could play a role as reservoir for urban cycle disease transmission.

Therefore, we undertook a study of the sylvatic cycle zoonotic pathogens that can threaten humans in Zambia. The pathogens tested here included: *Anaplasma* spp., *Trypanosoma* spp*., Rickettsia* spp*., Coxiella burnetii*, *Leishmania* spp., *Babesia* spp*., Plasmodium* spp*., Ehrlichia* spp*.* and *Borrelia* spp. in African NHPs.

## Methods

### Sample collection and DNA extraction

**Figure 1:**
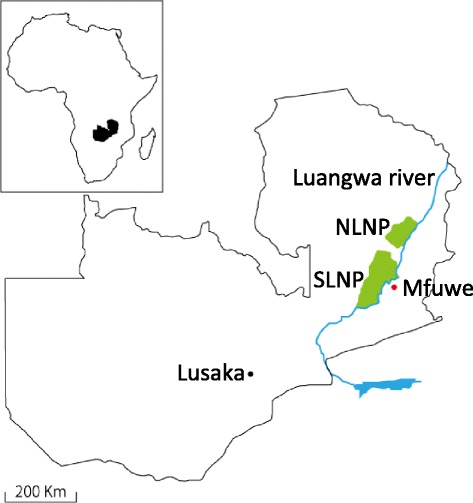
**Map of Zambia showing the sampling site.** NLNP: North Luangwa National Park, SLNP: South Luangwa National Park of the Luangwa valley ecosystem a Human African Trypanosomiasis (HAT) focus.

Spleen samples were obtained from 48 yellow baboons (*Papio cynocephalus*) and 40 vervet monkeys (*Chlorocebus pygerythrus*) in 2008. The sampling was conducted at Mfuwe in South Luangwa National park, Zambia (13°14′42.00′′ S, 31°38′54.07′′ E) (Figure 1). Eighty eight spleen DNA samples were analyzed in the current study for *Anaplasma* spp., *T. brucei rhodesiense* and *T. brucei gambiense*, *Rickettsia* spp*.*, *Coxiella burnetii*, *Leishmania* spp., *Plasmodium* spp*., Babesia* spp., and *Borrelia* spp. DNA was extracted from these organs by using the QIAamp DNA Mini Kit (Qiagen, Valencia, CA) according to the manufacturer’s instructions.

#### Ethical clearance

The culling was conducted under the permission from the Zambia Wildlife Authority (ZAWA) and the Institutional Ethical and Animal Care guidelines were adhered to during the culling and sampling exercise.

### Molecular identification of pathogens

#### PCR amplifications

PCR reactions were conducted using Amplitaq Gold® 360 reagent (Applied Biosystems, Foster City, CA) in a 20 μl reaction volume. All the primer sets employed in this study and PCR conditions can be found in Table 1 [8-15]. The PCR products were electrophoresed in a 1.5% agarose gel stained with Gel-Red^™^ (Biotium, Hayward, CA) and were visualized under UV light.

**Table 1:** **Primers and conditions for PCR detection of pathogen DNA**

**Organism**	**Target gene**	**Primer**	**Sequence (5' to 3')**	**Amplicon**	**Annealing temperature (°C)**	**Reference**
				**size (bp)**		
Rickettsia spp	*gltA*	RpCS.780p	GACCATGAGCAGAATGCTTCT	600	48	[8]
		RpCS.877p	GGGGACCTGCTCACGGCGG	480	54	
		RpCS.1273r	CATAACCAGTGTAAAGCTG			
Anaplasma sp..	16S rDNA	EHR16SD	GGTACCYACAGAAGAAGTCC	345	53	[9]
		EHR16SR	TAGCACTCATCGTTTACAGC			
*Coxiella burnetii*	IS1111	Trans 1	TATGTATCCACCGTAGCCAGTC	687	60	[10]
		Trans 2	CCCAACAACACCTCCTTATTC			
*Borrelia* spp*.*	fla gene	BflaPAD	GATCA(G/A)GC(T/A)CAA(C/T)ATAACCA(A/T)ATGCA		55	[11]
		BflaPDU	AGATTCAAGTCTGTTTTGGAAAGC			
		BflaPBU,nest	GCTGAAGAGCTTGGAATGCAACC	340	55	
		BflaPCR,nest	TGATCAGTTATCATTCTAATAGCA			
*B. microti*	18S rDNA	Babl	CTTAGTATAAGCTTTTATACAGC	238	55	[12]
		Bab4	ATAGGTCAGAAACTTGAATGATACA			
*Trypanosoma* spp*.*	ITS1 rDNA	ITS1 CF	CCGGAAGTTCACCGATATTG	Variable	58	[13]
		ITS1 BR	TTGCTGCGTTCTTCAACGAA			
*Leishmania* spp*.*	kDNA minicircle	L.MC-1S	CTRGGGGTTGGTGTAAAATAG-	700	55	[14]
		L.MC-1R	TWTGAACGGGRTTTCTG			
*Plasmodium* spp*.*	Cytb	DW2 &DW4	DW2; TAATGCCTAGACGTATTCCTGATTATCCAG	1253	60	[15]
			DW4; TGTTTGCTTGGGAGCTGTAATCATAATGTG			
		Cytb1 & Cytb2	CYTb1; CTCTATTAATTTAGTTAAAGCACA	939	50	
			Tb2; ACAGAATAATCTCTAGCACC			

#### Sequencing

The amplified PCR products for *Babesia* spp. *Rickettsia* spp. and *Anaplasma* spp. were subjected to direct sequencing and phylogenetic analysis. The amplicons were treated with ExoSAP-IT (USB Corporation, Cleveland, OH). The sequencing reaction was carried out with the BigDye terminator kit version 3.1 and resolved with a 3130 ABI (Applied Biosystems) capillary sequencer. The DNA sequences obtained were submitted to the DNA Data Bank of Japan (DDBJ) (http://www.ddbj.nig.ac.jp) under accession nos. AB844434 to AB844437. Phylogenetic analysis of the pathogens (*R. africae*:A, 426 bp, 16S rRNA; *A. phagocytophilum*:B, 345 bp 16S rRNA and *B. microti*:C, 238 bp, 18S rRNA) detected in primates from Zambia was based on 16S rRNA or 18S rRNA sequences respectively. The tree was constructed using the neighbor-joining method and ClustalW alignment.

### Statistical analysis

Statistical analysis of prevalence data was done using Chi-square statistical test. The chi-square test was meant to test the null hypothesis, which states that there is no significant difference between the expected and observed result.

## Results

PCR assays using genus- or species-specific primers for the selected zoonotic pathogens detected *Rickettsia* spp*.* in 35 samples (39.8%), *Anaplasma* spp. in 12 samples (13.6%) and *Babesia* spp. in 2 samples (2.3%). However, *Borrelia* spp., *Trypanosoma* spp., *Plasmodium* spp*., Leishmania* spp., and *Coxiella burnetii* were not detected. Important to note, *Babesia* spp. was only detected in baboons (Table 2). There was no significant difference between the infection prevalence, primate species and sex of the primates as tested by Chi square test (data not shown). Information on age of the primates was not available.

**Table 2:** **The prevalence of zoonotic pathogens in non-human primates in Zambia**

	**Baboon (n = 48)**			**Vervet monkey (n = 40)**		
	**Sex**		**Sub-total (%)**	**Sex**		**Sub-total (%)**
	**M (39)**	**F (9)**		**M (33)**	**F (7)**	
*Anaplasma* spp*.*	3	2	5 (10.4%)	6	1	7 (17.5%)
*Babesia* spp*.*	1	1	2 (4.2%)	0	0	0
*Borrelia* spp*.*			0			0
*Coxiella burnetii*			0			0
*Leishmania* spp*.*			0			0
*Plasmodium* spp*.*			0			0
*Rickettsia* spp*.*	14	2	16 (33.3%)	15	4	19 (47.5%)
*Trypanosoma* spp*.*			0			0

Some of the positive samples with genus-specific primers were further subjected to direct sequencing and the BLAST sequence homology searches were performed. Two *Rickettsia* spp.-positive samples had 99% identity with *R. africae* from Nigerian ticks [16]. Two *Anaplasma* spp*.*-positive samples were sequenced and showed 100% similarity with *A. phagocytophilum* from various host species and geographical regions. Two *Babesia* spp.-positive samples from baboons showed the highest sequence similarity with *Babesia* spp. KMG-2009a from baboons with 100% identities, and also showed 98% similarity with *B. leo*-K8, the isolate from a domestic cat in South Africa, and 99% similarity with *Babesia* spp. from a laboratory raised baboon in USA [17].

## Discussion

An investigation of pathogens in wild NHPs found in habitats close to human settlements is of importance in the control and eradication of probable human zoonotic pathogens.

**Figure 2:**
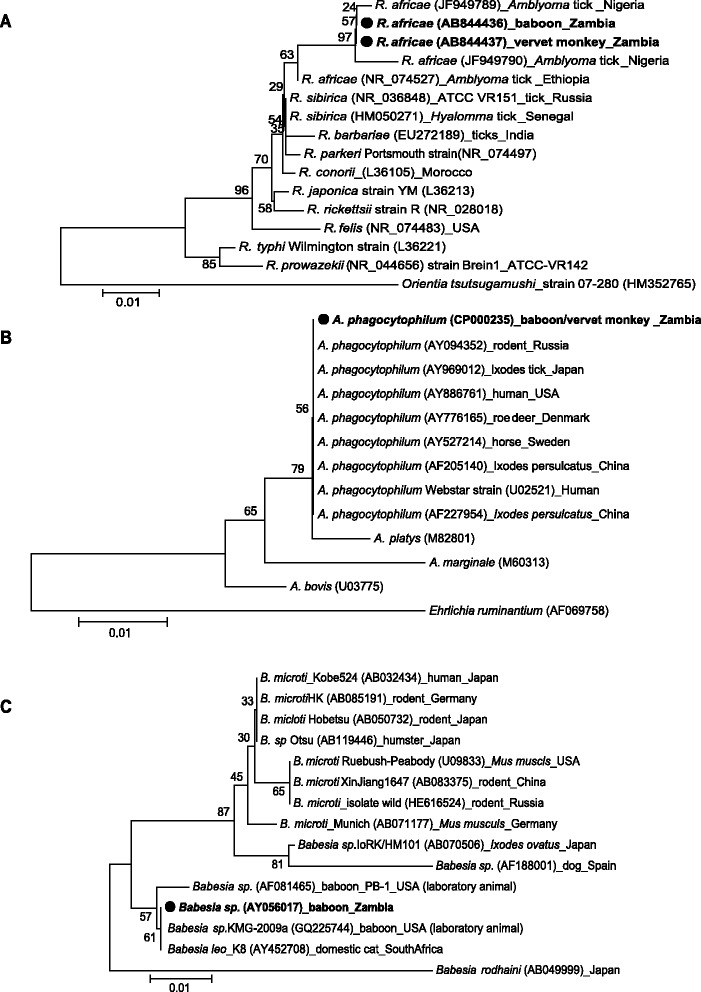
**Phylogenetic positions of the pathogens (** ***R. africae*** **: A, 426 bp, 16S rRNA;** ***A.*** ***phagocytophilum:*** **B**, **345bp 16S rRNA and** ***B. microti*** **C,** **238bp, 18S rRNA) detected in primates from Zambia based on 16S rRNA or 18S rRNA sequences respectively.** The tree was constructed using the neighbor-joining method and ClustalW alignment, and numbers on the tree indicate 1000 bootstrap values for branch points. Accession numbers are indicated.

We detected *Rickettsia* spp. in a total of 35 samples (39.8%). Further sequencing analysis revealed that some of the sequences were highly similar to that of *R. africae* (Figure 2). This is an agent of African tick bite fever, an acute and flu-like illness that is frequently accompanied by severe headache, inoculation eschars with regional lymphadenitis, vesicular cutaneous rash, and aphthous stomatitis [18,19]. The disease is transmitted in rural sub-Saharan Africa by ungulate ticks of the *Amblyomma* genus, mainly *Amblyomma hebraeum* in southern Africa and *Amblyomma variegatum* in west, central, and east Africa [20]. Phylogenetic comparisons between our obtained sequence and previous studies worldwide revealed a close relationship between Zambian and Nigerian *R. africae* isolates, suggesting general occurrence of rickettsioses in African continent.

We also obtained the sequences associated with *A. phagocytophilum* from both baboons and vervet monkeys (Figure 2). *A. phagocytophilum*, an obligate intracellular bacterium, is the agent of human granulocytic anaplasmosis, formerly known as human granulocytic ehrlichiosis [21]. This bacterium can infect humans and numerous animal species, including horses, cats, dogs, ruminants, and wildlife. In our analysis of *A. phagocytophilum* 16S rRNA gene, we found that the sequences of 16S rRNA were very conserved not only between African isolates but also between the other isolates of world-wide origin and this was in agreement with previous studies [22].

The rodent parasite *B. microti* and the bovine pathogen *Babesia divergens* appear to be responsible for virtually all of the known human zoonotic *Babesia* cases [23,24]. We detected a *B. microti-*like parasite from Zambian primates at a prevalence of 2.3% from baboons. Because *B. microti* shares a vertebrate host reservoir, the white-footed mouse (*Peromyscus leucopus*) and tick vector (*Ixodes dammini*) with *B. burgdorferi*, it might be expected that the caseload for human babesiosis will parallel the rise in the number of cases of Lyme disease in endemic areas [25-27].

*Babesia microti*, long considered on morphological grounds to be a single species found only in rodents, is now thought to consist of a complex of closely related subspecies, many of which are found in non-rodent hosts. Goethert and Telford III [28] identified 3 clades based on analysis of the 18S rRNA and beta-tubulin genes, with one (Clade 1) containing the majority of strains thought to be zoonotic. This clade includes the American zoonotic strains that have caused most babesiosis cases worldwide, but there are also separate zoonotic strains occurring in Japan (‘Kobe’ and ‘Hobetsu’) and Taiwan [29]. Strains of unknown zoonotic potential but closely related to the zoonotic American strains, according to 18S rRNA or beta-tubulin gene analysis, have been isolated in Germany (Hannover), central and eastern Russia (Mis, near Berezniki, Perm region and Vladivostok), Japan, South Korea and north-east China (Xinjiang) [28,30-32]. The zoonotic potential of Zambian *B. microti*-like parasite found calls for further investigation.

The genus *Borrelia* comprises of 37 known species of which 12 species are known to cause Lyme disease and are transmitted by ticks. *Borrelia burgdorferi* sensu *lato* complex, which is related to Lyme disease, is classified into four genospecies on the basis of genetic, phenotypic, and immunological properties [33]. The endemic tick-borne relapsing fever spirochetes are transmitted through the bites of soft ticks of the genus *Ornithodoros*; *O. sonrai* serves as the principle vector for *Borrelia crocidurae* in West Africa, and *O. moubata* complex ticks effectively maintain these spirochetes in East Africa [34]. *Borrelia recurrentis* causes louse-borne relapsing fever and *B. duttonii* is the agent of East African tick-borne relapsing fever [35]. Some cases of Lyme disease have been reported in Kenya [36], but any *Borrelia* species were not detected in our study.

Although we have included several other pathogens, which have the potential for causing zoonoses, we could not detect those species in this study. Trypanosomes infect a wide range of wildlife species that constitute a reservoir of infection for both people and domestic animals. In Zambia, human African trypanosomiasis, caused by *T. brucei rhodesiense*, is endemic especially alongside the Luangwa Valley ecosystem [37]. However, active trypanosome infection was not demonstrated in sampled NHPs in our study, although the Luangwa Valley ecosystem is an active trypanosomiasis endemic focus with several human cases having been reported from the same area [38], and vervet monkeys are experimentally susceptible to African salivarian trypanosomes *T. b. rhodesiense* [39,40] and *T. b. gambiense* [41].

*Plasmodium* spp*.* was not detected in the current study. So far, the transmission of *P. knowlesi*, a malaria parasite of Southeast Asian macaques occurs from monkeys to humans in South-East Asia [42]. Coexistence of humans and monkeys in the same habitat has been driven in some cases by ecological conditions as observed in the transmission of *P. knowlesi* to the human population in Southeast Asia [43]. Recently, several additional *Plasmodium* species such as *P. cynomologi*, *P. inui*, *P. simium*, and *P. brasilianum* have been considered to be the zoonotic parasites from monkey to human, but none of them have been reported in Africa. Therefore, several authors hypothesized that monkeys may act as reservoirs for human malaria or vice versa [43]. In the wild, baboons harbour parasites closely related to *Plasmodium*, such as *Hepatocystis* spp., but they are not naturally susceptible to *Plasmodium* [44].

To the best of our knowledge, this is the first report of these potential zoonotic pathogens detected in non-human primates in Zambia. Therefore, zoonotic infections namely: human Babesiosis, Anaplasmosis and Rickettsiosis are suspected to be endemic in Zambia in humans and cases could be simply misdiagnosed especially as malaria due to the febrile nature of the illnesses.

## Conclusion

Our study revealed that, potential zoonotic pathogens; *R. africae*, *A. phagocytophilum* and *B. microti*-like parasites exist in Zambian non-human primates. Zoonosis transmission involves interplay between humans, livestock and wildlife, making disease control complicated due to the lack of knowledge of the roles played by each. Better understanding of zoonotic pathogens harbored in non-human primates is necessary and must be adopted as a control measure in all regions inhabited by these animals or where they are in close proximity with human beings.

## Abbreviations

EID: Emerging infectious diseases
NHP: Non-human primates
ZAWA: Zambia Wildlife Authority
TG: Typhus group
SFG: Spotted fever group

## References

[1] Kooriyama T, Okamoto M, Yoshida T, Nishida T, Tsubota T, Saito A, Tomonaga M, Matsuzawa T, Akari H, Nishimura H, Miyabe-Nishiwaki T (2013). Epidemiological study of zoonoses derived from humans in captive chimpanzees. Primates.

[2] Bekker JL, Hoffman LC, Jooste PJ (2012). Wildlife-associated zoonotic diseases in some southern African countries in relation to game meat safety: A review. Onderstepoort J Vet Res.

[3] Kaiser J (2003). Conservation biology. Ebola, hunting push ape populations to the brink. Science.

[4] Whitfield J (2003). The law of the jungle. Nature.

[5] Kondgen S, Kuhl H, N’Goran PK, Walsh PD, Schenk S, Ernst N, Biek R, Formenty P, Matz-Rensing K, Schweiger B, Junglen S, Ellerbrok H, Nitsche A, Briese T, Lipkin WI, Pauli G, Boesch C, Leendertz FH (2008). Pandemic human viruses cause decline of endangered great apes. Curr Biol.

[6] Chomba C, Senzota R, Chabwela H, Mwitwa J, Nyirenda V (2012). Patterns of human – wildlife conflicts in Zambia, causes, consequences and management responses. J Ecol Nat Environ.

[7] Lloyd-Smith JO, George D, Pepin KM, Pitzer VE, Pulliam JRC, Dobson AP, Hudson PJ, Grenfell BT (2009). Epidemic dynamics at the human-animal interface. Science.

[8] Ishikura M, Ando S, Shinagawa Y, Matsuura K, Hasegawa S, Nakayama T, Fujita H, Watanabe M (2003). Phylogenetic analysis of spotted fever group rickettsiae based on gltA, 17-kDa, and rOmpA genes amplified by nested PCR from ticks in Japan. Microbiol Immunol.

[9] Parola P, Roux V, Camicas JL, Baradji I, Brouqui P, Raoult D (2000). Detection of *Ehrlichiae* in African ticks by polymerase chain reaction. Trans R Soc Trop Med Hyg.

[10] Parisi A, Fraccalvieri R, Cafiero M, Miccolupo A, Padalino I, Montagna C, Capuano F, Sottili R (2006). Diagnosis of *Coxiella burnetii*-related abortion in Italian domestic ruminants using single-tube nested PCR. Vet Microbiol.

[11] Takano A, Goka K, Une Y, Shimada Y, Fujita H, Shiino T, Watanabe H, Kawabata H (2010). Isolation and characterization of a novel *Borrelia* group of tick-borne *borreliae* from imported reptiles and their associated ticks. Environ Microbiol.

[12] Persing DH, Mathiesen D, Marshall WF, Telford SR, Spielman A, Thomford JW, Conrad PA (1992). Detection of *Babesia microti* by polymerase chain reaction. J Clin Microbiol.

[13] Njiru ZK, Constantine CC, Guya S, Crowther J, Kiragu JM, Thompson RCA, Da´vila AMR (2005). The use of ITS1 rDNA PCR in detecting pathogenic African trypanosomes. Parasitol Res.

[14] Kato H, Uezato H, Gomez EA, Terayama Y, Calvopiña M, Iwata H, Hashiguchi Y (2007). Establishment of a mass screening method of sand fly vectors for *Leishmania* infection by molecular biological methods. Am J Trop Med Hyg.

[15] Prugnolle F, Durand P, Neel C, Ollomo B, Ayala FJ, Arnathau C, Etienne L, Mpoudi-Ngole E, Nkoghe D, Leroy E, Delaporte E, Peeters M, Renaud F (2010). African great apes are natural hosts of multiple related malaria species, including *Plasmodium falciparum*. Proc Natl Acad Sci U S A.

[16] Ogo NI, de Mera IG, Galindo RC, Okubanjo OO, Inuwa HM, Agbede RI, Torina A, Alongi A, Vicente J, Gortázar C, de la Fuente J (2012). Molecular identification of tick-borne pathogens in Nigerian ticks. Vet Parasitol.

[17] Bronsdon MA, Homer MJ, Magera JMH, Harrison C, Andrews RG, Bielitzki JT, Emerson CL, Persing DH, Fritsche TR (1999). Detection of enzoonotic babesiosis in baboons (*Papio cynoephalus*) and phylogenetic evidence supporting synonymy of the genera *Entopolypoides* and *Babesia*. J Clin Microbiol.

[18] Raoult D, Fournier PE, Fenollar F, Jensenius M, Prioe T, de Pina JJ, Caruso G, Jones N, Laferl H, Rosenblatt JE, Marrie TJ (2001). *Rickettsia africae*, a tick-borne pathogen in travelers to sub-Saharan Africa. N Engl J Med.

[19] Kelly P, Matthewman L, Beati L, Raoult D, Mason P, Dreary M, Makombe R (1992). African tick-bite fever: a new spotted fever group rickettsiosis under an old name. Lancet.

[20] Kelly PJ, Beati L, Mason PR, Matthewman LA, Roux V, Raoult D (1996). *Rickettsia africae* sp. nov., the etiological agent of African tick bite fever. Int J Syst Bacteriol.

[21] Dumler JS, Barbet AF, Bekker CP, Dasch GA, Palmer GH, Ray SC, Rikihisa Y, Rurangirwa FR (2001). Reorganization of genera in the families *Rickettsiaceae* and *Anaplasmataceae* in the order *Rickettsiales*: unification of some species of *Ehrlichia* with *Anaplasma, Cowdria* with *Ehrlichia* and *Ehrlichia* with *Neorickettsia*, descriptions of six new species combinations and designation of *Ehrlichia equi* and 'HGE agent' as subjective synonyms of *Ehrlichia phagocytophila*. Int J Syst Evol Microbiol.

[22] Zhang L, Wang G, Liu Q, Chen C, Li J, Long B, Yu H, Zhang Z, He J, Qu Z, Yu J, Liu Y, Dong T, Yao N, Wang Y, Cheng X, Xu J (2013). Molecular analysis of *Anaplasma phagocytophilum* isolated from patients with febrile diseases of unknown etiology in China. PLoS One.

[23] Piesman J (1987). Emerging tick-borne diseases in temperate climates. Parasitol Today.

[24] Telford SR, Gorenflot A, Brasseur P, Spielman A, Kreier J (1993). Babesial infections in humans and wildlife. Parasitic Protozoa.

[25] Benach JL, Coleman JL, Habicht GS, McDonald A, Grundwaldt E, Giron JA (1985). Serological evidence for simultaneous occurrences of Lyme disease and babesiosis. J Infect Dis.

[26] Dammin GJ, Spielman A, Benach JL, Piesman J (1981). The rising incidence of clinical *Babesia microti* infection. Hum Pathol.

[27] Spielman A (1976). Human babesiosis on Nantucket Island: transmission by nymphal *Ixodes* ticks. Am J Trop Med Hyg.

[28] Goethert HK, Telford SR (2003). What is *Babesia microti*?. Parasitol.

[29] Shih CM, Liu LP, Chung WC, Ong SJ, Wang CC (1997). Human babesiosis in Taiwan: asymptomatic infection with a Babesia microti-like organism in a Taiwanese woman. J Clin Microbiol.

[30] Zamoto A, Tsuji M, Kawabuchi T, Wei Q, Asakawa M, Ishihara C (2004). US- type *Babesia microti* isolated from small wild mammals in Eastern Hokkaido, Japan. J Vet Med Sci.

[31] Zamoto A, Tsuji M, Wei Q, Cho SH, Shin EH, Kim TS, Leonova GN, Hagiwara K, Asakawa M, Kariwa H, Takashima I, Ishihara C (2004). Epizootiologic survey for *Babesia microti* among small wild mammals in north eastern Eurasia and a geographic diversity in the beta-tubulin gene sequences. J Vet Med Sci.

[32] Gray JS (2006). Identity of the causal agents of human babesiosis in Europe. Int J Med Microbiol.

[33] Masuzawa T, Suzuki H, Kawabata H, Ishiguro F, Takada N, Yano Y, Yanagihara Y (1995). Identification of spirochetes isolated from wild rodents in Japan as *Borrelia japonica*. J Clin Microbiol.

[34] Cutler SJ, Abdissa A, Trape J-F (2009). New concepts for the old challenges of African relapsing fever borreliosis. Clin Microbiol Infect.

[35] Cutler SJ, Bonilla EM, Singh RJ (2010). Population structure of East African relapsing fever *Borrelia spp*. Emerg Infect Dis.

[36] Jowi JO, Gathua SN (2005). Lyme disease: report of two cases. East Afr Med J.

[37] Anderson NE, Mubanga J, Fevre EM, Picozzi K, Eisler MC, Thomas R, Welburn SC (2011). Characterisation of the wildlife reservoir community for human and animal trypanosomiasis in the Luangwa Valley, Zambia. PLoS Negl Trop Disease.

[38] Namangala B, Oparaocha E, Kajino K, Hayashida K, Moonga L, Inoue N, Suzuki Y, Sugimoto C (2013). Preliminary investigation of trypanosomosis in exotic dog breeds from Zambia's Luangwa and Zambezi Valleys using LAMP. Am J Trop Med Hyg.

[39] Ngure RM, Ndungu JM, Ngotho JM, Nancy MK, Maathai RG, Gateri LM (2008). Biochemical changes in the plasma of vervet monkeys (*Chlorocebus aethiops*) experimentally infected with *Trypanosoma brucei rhodesiense*. J Cell Anim Biol.

[40] Thuita JK, Kagira JM, Mwangangi D, Matovu E, Turner CMR, Masiga D (2008). *Trypanosoma brucei rhodesiense* transmitted by a single tsetse fly bite in vervet monkeys as a model of human African trypanosomiasis. PLoS Negl Trop Dis.

[41] Abenga JN, Anosa VO (2005). Serum total proteins and creatinine levels in experimental gambian trypanosomosis of vervet monkeys. Afr J Biotechnol.

[42] Cox-Singh J (2012). Zoonotic malaria: *Plasmodium knowlesi*, an emerging pathogen. Curr Opin Infect Dis.

[43] Yamasaki T, Duarte AMRC, Curado I, Summa MEL, Neves DVDA, Wunderlich G, Malafronte RS (2011). Detection of etiological agents of malaria in howler monkeys from Atlantic Forests, rescued in regions of Sa˜o Paulo city, Brazil. J Med Primatol.

[44] Garnham P (1966). Malaria Parasites and Other Haemosporidia.

